# Comparative study of K-wire combined with screw vs. K-wire in the treatment of AO type B3.1 phalangeal fractures

**DOI:** 10.1186/s12891-023-06731-0

**Published:** 2023-07-19

**Authors:** Xuelin Ma, Li Wang, Xiaoran Zhang, Zhemin Zhang, Yali Xu, Li Lv, Xinzhong Shao

**Affiliations:** Department of Hand Surgery, The 3rd Hospital, Hebei Medical University, NO. 139 Ziqiang Road, Shijiazhuang, 050051 Hebei People’s Republic of China

**Keywords:** K-wire, Screw, Phalangeal fractures, Comparative study

## Abstract

**Purpose:**

The purpose of this study was to introduce the surgical method of K-wire combined with screw in the treatment of Arbeitsgemeinschaftfür Osteosynthesefragen (AO) type B3.1 phalangeal fractures and to compare its clinical, radiological and functional outcomes with K-wire fixation.

**Methods:**

This was a retrospective comparative study. From January 2015 to February 2022, we treated 86 patients with AO type B3.1 phalangeal fractures. A total of 71 patients were finally included in the statistical analysis. Thirty-nine patients received K-wires combined with screw, and 32 patients received simple K-wires. The follow-up time was at least 6 months. Outcome measures included general information, operative time, total active motion (TAM), pinch strength, radiological union time, pain assessed by visual analog scale (VAS), Quick Disabilities of the Arm, Shoulder, and Hand (QuickDASH) score, cost, and complications.

**Results:**

The follow-up time was 6–12 months, with an average of 7.9 months. All patients achieved clinical and radiological union. Compared with the K-wire fixation group, the TAM, radiological union time and VAS score of the K-wire combined with screw group had obvious advantages. Compared with the opposite healthy hand, the grip strength of the two groups was similar, and there was no significant difference in the QuickDASH score. The incidence rate of complications in the K-wire combined with screw group (2/39) was lower than that in the K-wire fixation group (7/32).

**Conclusions:**

Compared with simple K-wire fixation, K-wire combined with screw in the treatment of AO type B3.1 phalangeal fractures is a safer and reliable surgical method. K-wire controls the rotation and plays a role similar to a “lock”. The screw can exert pressure and fix it more firmly. It shortens the time of fracture healing and has a higher TAM and fewer postoperative complications.

## Introduction

**Fig. 1:**
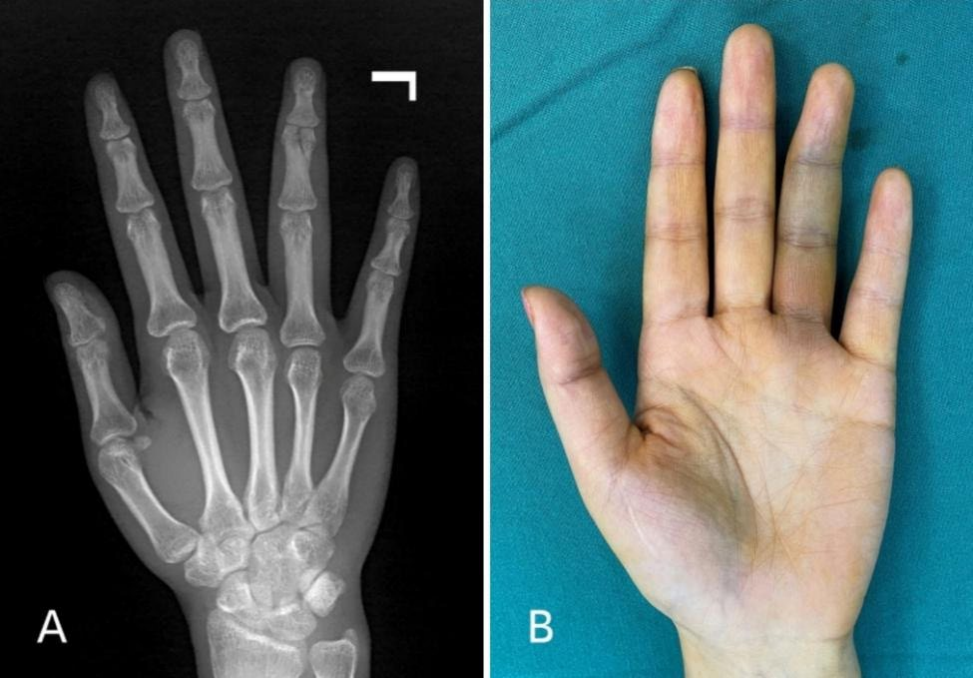
**a** The middle phalanx of left ring finger AO type B3.1 fracture. **b** The distal segment of the left ring finger deviates to the fracture site

AO type B3.1 phalangeal fracture is one of the common types [1]. This kind of fracture is characterized by a small fracture block. Due to the compression of the phalangeal base and the traction of the ligament, it is extremely unstable. The distal end of the finger deviates to the fracture site (Fig. 1), which mostly requires surgical treatment [2]. Traditional surgical methods include K-wire fixation, plate-screw internal fixation and simple screw fixation [3–6].

K-wire fixation is widely used in clinical work because of its relatively simple operation, reduced irritation to the tendon and reduced impact on the blood supply around the fracture. However, this method has poor pressure on fractures. In spiral, oblique and comminuted fractures, the reduction angle may be lost after operation [7], which often needs to be combined with postoperative splint external fixation. At the same time, the needle path is exposed for a long time, which is prone to infection, there is a potential risk of secondary operation, and the operation difficulty of the second operation increases. Although plate-screw internal fixation can achieve good functional recovery [8], plate-screw internal fixation requires extensive exposure, long operation time and difficult operation, which will produce many related risks of complications, including bone nonunion, infection, tendon stimulation and joint stiffness [9, 10]. This method increases the cost to patients and increases the burden at the same time. The screw play a role in fixation and compression in the treatment of oblique and spiral fractures of phalanges [11]. Limited incision and simple operation are some advantages of plate-screw fixation and make up for some of its shortcomings [12]. Therefore, we treated AO type B3.1 phalangeal fractures, and some patients used K-wire combined with screw technology. Taking advantage of the respective advantages of K-wire and screw, K-wire can reduce and prevent rotation, and the screw can pressurize the fracture. The tail of the K-wire is buried under the skin. Because of its firm fixation and anti rotation, active range of motion (ROM) exercises can be started on the second day after the operation.

In this study, we introduced the surgical method of K-wire combined with screw in the treatment of AO type B3.1 phalangeal fractures in detail. At the same time, a retrospective comparative study was used to compare the clinical, radiological and functional outcomes of K-wire combined with screw and K-wire in the treatment of AO type B3.1 phalangeal fractures. There has been no such comparative study in the past.

## Methods

This was a retrospective observational study approved by the ethics committee of The Third Hospital of Hebei Medical University. From January 2015 to February 2022, we treated 86 patients with AO type B3.1 phalangeal fractures. Finally, 71 cases were included in the statistical analysis. All operations were performed in the same department of hand surgery, and written informed consent was signed.

The inclusion criteria were as follows: patients aged 18 to 60 years; oblique fracture of AO type B3.1 of the proximal or middle phalanx from the index finger to the little finger; fracture displacement and articular surface collapse; acute fracture within 2 weeks; closed fracture or open small wound less than 1.5 cm; and healthy hand.

The exclusion criteria were as follows: combined with fractures in other parts of the hand; fracture with related tendon or nerve injury; surgical treatment of the hand; previous finger joint stiffness, arthritis, osteoporosis or immature bone development; diabetes, gout and other diseases that affect bone structure and joint activity; and loss to follow-up or refusal to participate.

### Surgical technique

All procedures were performed in an operating room under local, regional, or general anesthesia with the patient in the supine position. Hemostasis was performed with a finger tourniquet. If there was a small wound, it was closed after debridement.

### K-wire combined with screw

A lateral longitudinal incision of the finger was made on the fracture side, and the incision length accounted for approximately 2/3 of the longitudinal length of the fracture. The extensor tendon and the neurovascular bundle of the finger were separated to expose the phalanx and the collateral ligament of the interphalangeal joint. The finger was straightened, the distal finger of the fracture was pulled longitudinally, the fracture block was squeezed to the inside, and the fracture was reduced. The insertion point of the K-wire was selected at the dorsal edge of the collateral ligament of the interphalangeal joint to avoid penetrating the collateral ligament. A 0.8 or 1 mm K-wire was drilled into the fracture side of the phalangeal condyle through the guide. By comparison with adjacent fingers, no rotation and deflection deformities were checked, and the fracture reduction, length and position of the K-wire were confirmed under intraoperative fluoroscopy. After fracture reduction was good, at the proximal end of the insertion point of the K-wire (the proximal bone surface of the collateral ligament on the side of the interphalangeal joint), under the guidance of the guide, perpendicular to the direction of the fracture line, a 1.1 mm metal drill was used to drill and break through the contralateral cortex. The position of the drill bit was confirmed to be correct under intraoperative fluoroscopy. After measuring the length, a 1.5 mm full thread metal bone screw (Wego™, Shandong, China) was placed along the direction of the drill. If it was a long oblique fracture, a 0.8 or 1 mm K-wire could be drilled into the proximal end of the screw again. Finally, the length and position of the screw were confirmed through intraoperative fluoroscopy. The tip of the K-wire penetrated the contralateral cortex. The tail end of the K-wire was cut short and bent and buried under the skin on the side of the fingers. The incision was sutured with 5 − 0 Prolene suture. The incision was wrapped with sterile gauze without a finger splint.

### K-wire

The finger was straightened, the distal finger of the fracture was pulled longitudinally, and the fracture block was squeezed to the inside to realize closed reduction of the fracture. A 0.8 or 1 mm K-wire was drilled into the fracture side of the phalangeal condyle through the guide. By comparison with adjacent fingers, no rotation and deflection deformities were checked, and the fracture reduction, length and position of the K-wire were confirmed under intraoperative fluoroscopy. After confirmation, another 0.8 or 1 mm K-wire was drilled into the proximal or distal end of the first K-wire at different angles. If it was a long oblique fracture, cross drilling was continued into the third 0.8 or 1 mm K-wire. Finally, the length and position of the new K-wire were confirmed through intraoperative fluoroscopy. The drilling points of the K-wire were located on the side of the phalange between the extensor tendon and neurovascular system. The tip of the K-wire penetrated the contralateral cortex. The exposed end of the K-wire was cut short and bent 90°. After the sterile dressing was wrapped, the finger splint was used to fix the finger in a straight position.

### Postoperative management

In the K-wire combined with screw group, active ROM exercises were started on the second day after the operation. The dressing of the incision was changed once every 3–5 days until the wound healed and the suture was removed. X-ray examination was performed every 2 weeks after the operation. After fracture radiological union (usually 4–6 weeks after operation), passive exercise was added after active ROM exercise. The angle of passive exercise was based on the maximum tolerance and gradually increased. When the fracture was completely healed (usually 3 months after the operation), the K-wire and screw were removed under local outpatient anesthesia, and unrestricted activities were performed.

In the K-wire group, the finger splint was worn for 3 weeks. From 1 week after the operation, the finger splint was temporarily removed every day, and protected active ROM exercises were conducted. After 3 weeks, the finger splint was removed, and unrestricted active ROM exercises were initiated. X-ray examination was performed every 2 weeks after the operation. When radiography confirmed that the fracture healed and formed a callus, the K-wire was pulled out. When the fracture was completely healed (usually 3 months after operation), unrestricted activities were performed.

### Follow-up and postoperative evaluation

The shortest follow-up period was 6 weeks. Our main outcome measure was TAM. Secondary outcome measures included pinch strength, radiological union time, pain VAS score, QuickDASH score, cost, and the incidence of postoperative complications such as infection, reduction loss, nonunion, nerve injury and scar hyperplasia. X-ray examination was performed every 2 weeks after the operation until radiography showed that the fracture line disappeared and the bone cortex was continuous, which was considered fracture healing. There was no sign of fracture healing within 12 weeks, which was considered fracture nonunion. Postoperative pain was assessed by VAS. For pain intensity, the scale was anchored by no pain (score of 0) and worst imaginable pain (score of 100) [13]. We used a protractor to measure the active ROM of the metacarpophalangeal joint and interphalangeal joint of the affected finger and took the sum of them as the TAM. A Jamar dynamometer (Jamar, Preston, USA) was used to measure the finger pinch force. At the same time, to improve the consistency between the dominant side and the nondominant side, we defaulted that the pinch force on the dominant side was 6% higher than that on the nondominant side [14]. The QuickDASH questionnaire assessed subjective disability in arm and shoulder function, ranging from 0 (indicating no disability) to 100 (indicating maximum disability) [15]. The system retrieved the surgical costs of both groups of patients, and the K-wire and screw fixation group included the cost of surgical removal of the implant. During follow-up, complications were evaluated and recorded from the patient’s main complaint or the doctor’s examination.

### Statistical analysis

Continuous variables are represented as the mean and standard deviation (SD), and normality was tested by the Shapiro–Wilk test. Student’s t test was used for the difference between groups of variables conforming to a normal distribution, and the Mann–Whitney U test was used for variables not conforming to a normal distribution. Categorical variables were expressed in quantity and percentage, and the differences between groups were tested by the chi-square test or Fisher’s exact test. A p value less than 0.05 was considered to be statistically significant. All analyses were performed by SPSS Statistics Software version 25.0 (IBM corporation, Armonk, New York, USA).

## Results

Initially, we treated a total of 86 patients with AO type B3.1 phalangeal fractures, of which 5 patients were fixed with finger splints because of no displacement or refusal of operation, and 8 patients were excluded from the study due to tendon injury (n = 3), finger surgery before fracture (n = 1), finger movement disorder caused by joint stiffness or arthritis before fracture (n = 2), and chronic diseases that may affect bone healing and finger function (n = 2). Two patients were lost to follow-up due to geographical and work reasons. Finally, 71 patients (39 cases in the K-wire combined with screw group and 32 cases in the K-wire group) were included in the statistical analysis. We collected detailed clinical data of these 71 patients, including age, sex, injured hand side, handedness, fractured finger, fractured segment, and trauma etiology, which are shown in Table 1.

**Table 1:** Baseline demographic and clinical characteristics between two groups

Variable	Kirschner wire combined with screw (n = 39)	Kirschner wire cross fixation (n = 32)	p
Mean Age (y)	35.9(20–57)	34.3(19–59)	0.59
Gender (%)			0.81
Male	23 (59.0)	18(56.2)	
Female	16 (41.0)	14(43.8)	
Injured hand			0.93
Left	13 (33.3)	11(34.4)	
Right	26 (66.7)	21(65.6)	
Handedness(%)			0.28
Dominant	29 (77.4)	20(62.5)	
Non-dominant	10 (25.6)	12(37.5)	
Fractured finger(%)			0.9
Index	7(17.9)	10(31.2)	
Middle	11(28.2)	7(21.9)	
Ring	15(38.5)	12(37.5)	
Little	6(15.4)	3(9.4)	
Segment(%)			0.26
Proximal Segment	12(30.8)	14(43.7)	
Middle Segment	27(69.2)	18(56.3)	
Trauma etiology(%)			< 0.001
Falling	14 (35.9)	11(50.0)	
Twisting	12(30.8)	11(18.8)	
Blunt trauma	11(28.2)	9(28.1)	
Other	2 (5.1)	1(3.1)	

All patients were followed up for an average of 8 (6–12) months (P > 0.05). The average time from injury to surgery was 6 (1–12) days (P > 0.05). The operation time of the K-wire fixation group (mean 34.6 min) was shorter than that of the K-wire combined with screw group (mean 47.7 min) (P < 0.001). At the final follow-up, according to the results of statistical data analysis, TAM, radiological union time, and VAS in the K-wire combined with screw group were significantly better than those in the K-wire fixation group (P < 0.001). Grip strength compared with the opposite healthy hand and QuickDASH scores were similar between the two groups, and there was no significant difference in statistical results (P > 0.05). In terms of cost, the K-wire fixation group only required one operation, so it had a significant advantage (P < 0.001) (Table 2).

**Table 2:** Comparison of clinical, radiological, functional outcomes and complications between the two groups at the last visit

Variable	K-wire combined with screw n = 39	K-wire cross fixation n = 32	p
Mean time from injury to surgery(d)	6.4(2–10)	5.7(1–12)	0.24
Mean follow-up(mo)	7.8(6–12)	8.1(6–12)	0.42
Mean operative time(min)	47.7(35–57)	34.6(26–43)	< 0.001
Mean TAM	259.4(251–265)	223.7(214–245)	< 0.001
Mean pinch strength % of healthy side	95.6(92.3–98.6)	94.8(90.2–98.6)	0.18
Mean time to union(wk)	4.2(4.0–5.0)	5.2(4.0–6.0)	< 0.001
Mean pain assessment on VAS (0–10)	0.60(0–2)	1.2(0–4)	< 0.001
Mean quick DASH score (0–100)	6.1(2–22)	8.7(2–34)	0.661
Cost(US$) Overall complications	349(297–401) 2(5.1%)	611(531–700) 7(21.9%)	< 0.001
Infection	0	2	
Reduction loss	0	4	
Nonunion	0	0	
Nerve injury	0	1	
Scar hypertrophy	2	0	

Percentages show involved hand compared with opposite normal hand; TAM: We regard the sum of active ROM of the metacarpophalangeal joint and interphalangeal joint of the affected finger as TAM.

During the follow-up, the complications of the K-wire combined with screw group (2 out of 39, 5.1%) were less than those of the K-wire group (7 out of 32, 22%). In the K-wire combined with screw group, the complication in 2 cases was scar hypertrophy, which was alleviated by drug and physical therapy. In the K-wire group, 2 patients had K-wire infection, which showed that the skin around the K-wire was red, the skin temperature increased and secretions, but they were cured by dressing change and antibiotics; 4 patients had loss of reduction angle, 1 of which was caused by accidental pulling out of K-wire, but because the fracture displacement angle of 4 patients was small, they were treated by prolonging the external fixation time and giving up early active ROM exercise (exercise after radiological union); and 1 patient had symptoms of nerve injury and felt numb in the distal finger abdomen of the injured side. The nerve contusion was considered to be caused by repeated penetration of the K-wire. The patient completely recovered after 1 month through neurotrophic drugs and physical therapy. There was no fracture nonunion in either group.

**Fig. 2:**
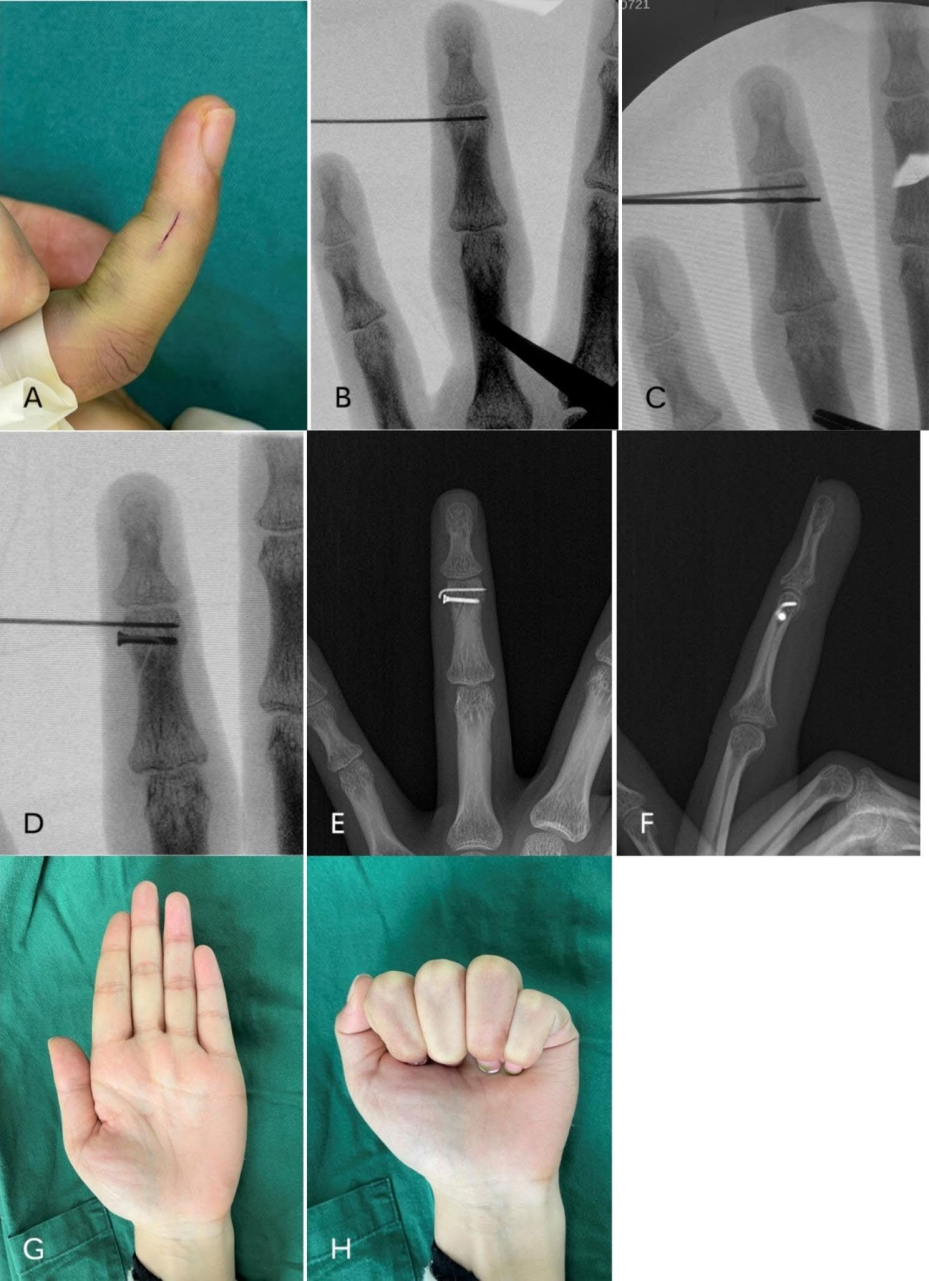
A 27-year-old female patient with an AO type B3.1 fracture of the middle phalanx of the left ring finger received K-wire combined with screw. **a** Micro incision on the side of finger fracture. **b** to **d** showed the operative process of K-wire combined with screw. **e**, **f** were the postoperative radiograph. **g**, **h** showed the functional outcome at 2 months

**Fig. 3:**
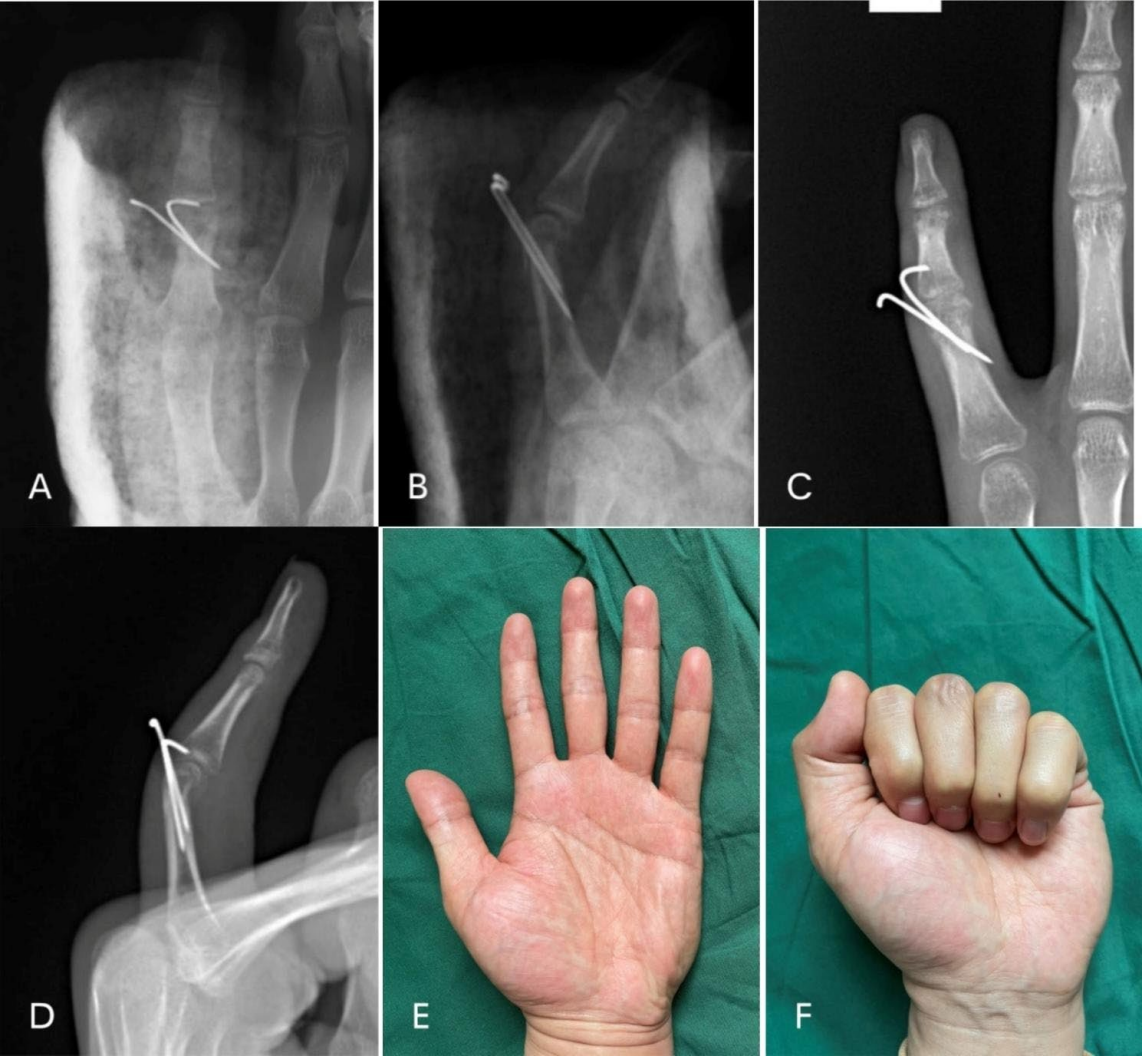
**A** 32-year-old male patient with an AO type B3.1 fracture of the proximal phalanx of the left little finger received K-wires fixation. **a**, **b** Two K-wires were used to fix the fracture and a plaster splint was used for external fixation. **c**, **d** showed the radiological findings 6 weeks after the operation. **e**, **f** showed the functional outcome at 2 months

Figures 1 and 2 presented 2 typical cases of AO type B3.1 phalangeal fractures received K-wires combined with screw (Fig. 2) or simple K-wires (Fig. 3). At the last visit, the ROM showed satisfactory results.

## Discussion

K-wire fixation is the most traditional surgical treatment for AO type B3.1 phalangeal fractures, but complications such as loss of reduction and needle infection are potential problems. Compared to simple K-wire fixation, K-wire combined with screw fixation can improve fracture union, obtain better finger function, alleviate postoperative pain, and reduce the incidence of postoperative complications.

**Fig. 4:**
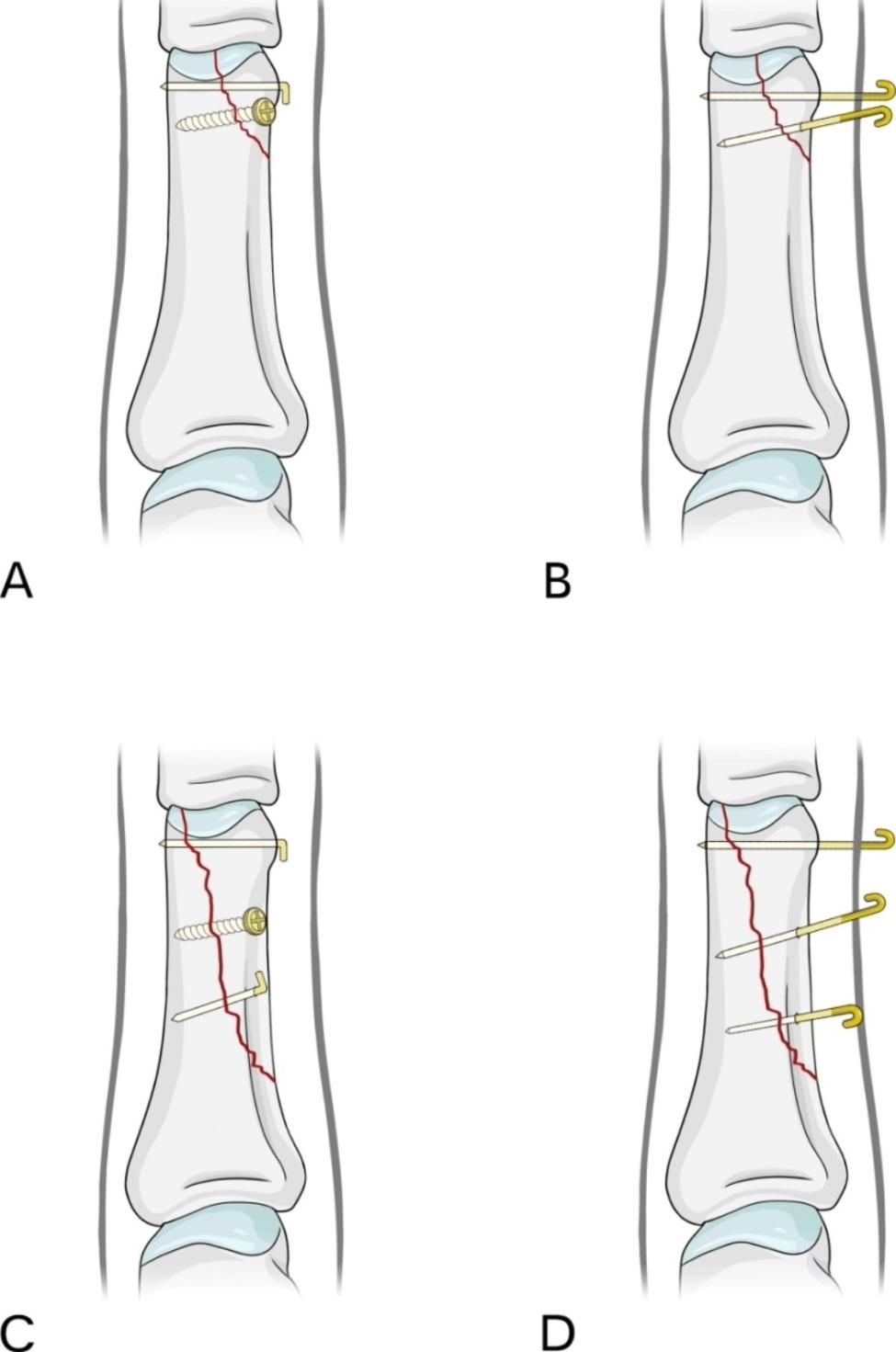
**a** K-wire combined with one screw. **b** Two K-wires. **c** Two K-wires combined with one screw. **d** Three K-wires

The AO classification of phalangeal fracture type B3 fracture is an intra-articular fracture of the distal part of the phalange, while type 1 of B3 refers to avulsion or splitting fracture [16]. The London classification of phalangeal condyle fracture in 1971 further divided this type of fracture. Type I: fracture involving 50% of the articular surface; Type II: fracture involving less than 50% of the articular surface; Type III: fracture involving more than 50% [17]. Although this type of fracture block is very small, because it is an intra-articular fracture, improper treatment may lead to important problems in patient satisfaction and function [11, 18]. When choosing treatment methods, fracture stability is an important indication. Stable fractures need conservative treatment, while unstable fractures need active surgical treatment [19–21]. Conservative treatment often leads to joint stiffness or malunion, resulting in partial loss of function [2]. AO type B3.1 phalangeal fractures are unstable fractures that should be treated surgically as much as possible. Firm fixation can carry out early ROM exercise and obtain good function [22, 23]. Therefore, we used K-wire combined with screw and simple K-wire fixation to treat AO type B3.1 phalangeal fractures. During the operation, short oblique fractures were fixed with one K-wire combined with one screw or two K-wires, and long oblique fractures were fixed with two K-wires combined with one screw or three K-wires (Fig. 4).

The average operation time of K-wire combined with screw fixation (47.7 min) was longer than that of K-wire fixation (34.6 min) (P < 0.001). Because the former needed the incision to expose the bone on the side of the finger, the entry point of the K-wire and the entry point of the screw were determined, and the drill bit needed to be positioned before the screw was screwed in. This process will take some time. K-wire fixation reduced the early process. The operations were performed by experienced surgeons. After closing and resetting, the number of repeated drillings was reduced as much as possible. However, because the K-wire fixation group closed drilling, although there was anatomical experience as a guide, it was still blind. In the K-wire fixation group, 5 cases had surgical procedures that exceeded the shortest surgical time in the K-wire combined with screw fixation group, all of which were related to the blindness of K-wire closed drilling. Previous literature reported that closed reduction and K-wire fixation were easily complicated with nerve injury [24]. One case of finger nerve injury occurred in our K-wire fixation group. It may be related to the excessive deviation of the drilling point of the Kirschner wire to the palmar side and the excessive drilling length. The VAS score of 0.60 (0–2) for K-wire combined with screw fixation was lower than that of 1.2 (0–4) for K-wire fixation (P < 0.001). ROM exercise could begin on the second day after surgery with K-wire and screw fixation. The K-wire fixation group routinely required splint fixation for three weeks after surgery. At the same time, the latter had a loss of reduction and infection, resulting in prolonged splint fixation time. The blindness of K-wire fixation could also stimulate joint ligaments and tendons, which may cause joint stiffness or adhesion. Although effective ROM exercise was performed after the removal of the splint, passive activity during reexamination could cause relatively severe pain. Therefore, we inferred that reducing joint stiffness and adhesion by shortening the time of splint fixation could improve postoperative pain. In the past, many studies reported that the exposure of K-wire would be associated with the risk of needle tract infection [25–27]. There was an article comparing external and buried K-wires. Exposed K-wires greatly increased the risk of infection [28]. There were 2 cases of infection in our K-wire fixation group, which verified the previous conclusions. Although the K-wire combined with screw fixation group needed to be incised, it did not irritate the tendons and nerves. Finally, the tail of the K-wire was cut short and buried in the subcutaneous soft tissue to reduce the risk of infection. Although secondary surgery was needed, it only needed to be treated under local outpatient anesthesia. High patient satisfaction.

In this study, the average healing time was 4.2 (4.0–5.0) weeks in the K-wire combined with screw group and 5.2 (4.0–6.0) weeks in the K-wire fixation group. In the previous literature, the average healing time of phalangeal fractures was 4–8 weeks [3]. The average TAM of the K-wire combined with screw group was 259.4 (251–265) degrees and that of the K-wire fixation group was 223.7 (214–245) degrees. In terms of healing time and TAM, the K-wire combined with screw group was statistically better than the K-wire fixation group (P < 0.001). K-wire controlled the rotation of the fracture and played a role similar to “lock”. The screw could exert pressure on the fracture and made the fixation more firm. Due to differences in the stability of the two fixation methods, the timing of early postoperative exercise is different. Ultimately, we attributed this result to periosteal repair and early activity [8, 29]. In our study, there were 2 cases of infection and 4 cases of loss of reduction. Long-term inflammatory effects and unstable fracture fixation were the reasons for the prolonged nonunion of phalangeal fractures. The long-term external fixation could lead to a decrease in TAM. However, it was gratifying to note that there was no case of nonunion in the end. After fracture healing, there was no statistically significant difference in finger pinch strength between the two groups (P > 0.05), which was related to effective exercise for both patients after surgery. The average operating cost for the K-wire and screw group was US$ 349 (297–401), and the average operating cost for the K-wire fixation group was US$ 611 (531–700). Usually, the price of a cement package is about US$50, and the price of each K-wire is from $3 (stainless steel) to US$ 45 (titanium alloy). The price of a metal screw is about US$ 100. At the same time, the combination of K-wire and screw fixation required a second surgical removal of the implant, which was relatively expensive compared to K-wire fixation. It was also a factor that we had to consider when choosing the surgical method.

There were several limitations to this study. First, the retrospective design was its limitation. Although no significant differences were observed in demographic and preoperative variables, there were inherent limitations in data collection due to non-randomization, which may lead to selection bias and ultimately lead to statistical analysis of clinical efficacy. Second, we could not examine the imaging parameters or functional status of patients before injury or the potential factors that may affect the recovery effect in the process of postoperative recovery. Third, the sample size was small, which may lead to type II statistical errors in evaluating some variables. Fourth, the operation was completed by a department but not a complete group of doctors. There may be selection preferences, which may affect the results. We believe that a prospective, randomized, controlled, and even multicenter study will be more helpful in our research on the treatment of this type of fracture.

In conclusion, K-wire combined with screw in the treatment of AO type B3.1 phalangeal fractures is a safe and reliable surgical method. Because of its anti-rotation and compression effect, the fixation is more reliable, supports early active ROM exercise, shortens the time of fracture healing, and has higher TAM and fewer postoperative complications. However, secondary surgery is required to remove the implant, so the clinical cost is high. These are all factors that we should consider in our treatment. In clinical practice, without considering secondary surgery and costs, K-wire combined with screws is a better choice for the treatment of AO type B3.1 phalangeal fractures.

## Data Availability

All the data will be available upon motivated request to the corresponding author of the present paper.

## List of abbreviations

AO: Arbeitsgemeinschaftfür Osteosynthesefragen
VAS: Visual analogue scale
QuickDASH: Quick Disabilities of the Arm, Shoulder, and Hand
TAM: Total active motion
ROM: Range of motion
SD: Standard deviation
